# Preoperative Six-Minute Walking Distance as a Predictor of Postoperative Complications in Patients Undergoing Lobectomy for Non-Small-Cell Lung Cancer

**DOI:** 10.3390/arm93060052

**Published:** 2025-11-24

**Authors:** Naoki Maki, Takahiro Yanagihara, Ashoka Indranatha Wijesinghe, Kazuto Sugai, Tomoyuki Kawamura, Yusuke Saeki, Shinsuke Kitazawa, Naohiro Kobayashi, Shinji Kikuchi, Yukinobu Goto, Harumi Sakamoto, Keisuke Taniguchi, Hideo Ichimura, Yukio Sato

**Affiliations:** 1 Faculty of Rehabilitation, R Professional University of Rehabilitation, 2-12-31 Kawaguchi, Tsuchiura 300-0032, Ibaraki, Japan; 2 Department of Thoracic Surgery, Faculty of Medicine, University of Tsukuba, 1-1-1 Tennodai, Tsukuba 305-8577, Ibaraki, Japan; 3 Department of Thoracic Surgery, Tokyo Medical University Ibaraki Medical Center 3-20-1 chuo, Ami-machi, Inashiki-gunn 300-0395, Ibaraki, Japan

**Keywords:** video-assisted thoracic surgery, lung cancer, postoperative complications, 6-minute walk test, preoperative assessment, risk prediction

## Abstract

**Highlights:**

**What are the main findings?**

- Preoperative six-minute walking distance (6MWD) of ≤450 m was independently associated with a 5.6-fold higher risk of 30-day postoperative pulmonary complications after VATS lobectomy for non-small-cell lung cancer.
- In the logistic regression analysis focusing on pulmonary complications, the length of hospital stay was further identified as a significant factor.

**What are the implications of the main findings?**

- A simple, low-cost six-minute walk test (6MWT) can serve as a practical preoperative risk stratification tool, complementing routine spirometry to identify high-risk patients.
- Patients with a 6MWD of ≤450 m may benefit from targeted prehabilitation or intensified perioperative management to reduce postoperative morbidity.

**Abstract:**

Introduction: Minimally invasive video-assisted thoracic surgery (VATS) for lung cancer has become a widely used approach. However, postoperative pulmonary complications (PCs) such as pneumonia, atelectasis, and lung fistula remain significant challenges, particularly in older adult patients with multiple comorbidities. The 6-minute walk test (6MWT) has been suggested as a predictor of postoperative outcomes in various surgical settings, but its relationship with postoperative complications following VATS lobectomy for lung cancer has not been thoroughly explored. The aim of this study was to determine if preoperative 6MWD predicted the occurrence of 30-day PCs among patients undergoing VATS lobectomy for non-small-cell lung cancer. Methods: This retrospective study examined 66 patients who underwent VATS lobectomy for lung cancer. Participants were categorized into two groups: those with postoperative pulmonary complications (*n* = 11) and those without (*n* = 55). The research period was from January to September 2022. The preoperative 6MWT distance, along with other clinical and demographic factors, was assessed to determine its predictive value for postoperative complications. Multivariate logistic regression analysis was performed to identify significant predictors. Results: The study found that preoperative 6MWT ≤ 450 m was a significant predictor of postoperative pulmonary complications (odds ratio: 5.674, 95% CI: 1.206–26.684, *p* = 0.028). Conclusions: The preoperative 6MWT distance is a useful predictor of postoperative pulmonary complications in patients undergoing VATS lobectomy for lung cancer. Patients with a 6MWT ≤ 450 m may be at higher risk for complications such as pneumonia, atelectasis, and lung fistula. Incorporating preoperative 6MWT as a risk stratification tool could help guide clinical decisions and rehabilitation efforts to improve postoperative outcomes in this patient population.

## 1. Introduction

In recent years, less invasive surgical approaches, video-assisted thoracic surgery (VATS) and robotic-assisted thoracic surgery (RATS), have been increasingly adopted for lung cancer resection because they enable smaller incisions, reduce surgical stress, and facilitate faster postoperative recovery, with the expectation of fewer postoperative complications (PCs) [1,2]. In Japan, this shift toward minimally invasive thoracic procedures is evident, with VATS lobectomy accounting for approximately 80–90% of all lobectomies in 2023, in line with a broader national trend toward less invasive care [3].

Despite these advances, many patients who undergo lung cancer surgery are older adults and have multiple comorbidities, factors that inherently elevate the risk of PCs and may attenuate the benefits of minimally invasive techniques [4]. Contemporary risk models also indicate the persistent burden of postoperative morbidity following lung cancer resection, with consequences that include prolonged hospitalization, delayed recovery, and potential disruption of adjuvant therapy delivery [5]. Among the mechanisms implicated in respiratory PCs, delayed mobilization and impaired sputum expectoration are key contributors [6,7]. Anesthesia and surgical stress can transiently reduce cough effectiveness and respiratory muscle performance, while postoperative pain and bed rest hinder airway clearance; together, these factors may promote bronchial obstruction, atelectasis, or infection [7,8,9,10].

Preoperative physical function, particularly exercise tolerance, therefore, represents a clinically meaningful dimension of perioperative risk. Patients with better fitness are more likely to mobilize early and clear secretions effectively, potentially reducing the incidence of respiratory PCs. Professional guidelines emphasize fitness assessment when considering radical therapy in lung cancer, reinforcing the rationale for integrating functional measures into preoperative evaluation [6]. Within this context, the six-minute walk test (6MWT) is a simple, non-invasive measure of submaximal aerobic capacity that can be performed in standard clinical settings and may serve as an actionable indicator for perioperative stratification.

The present study investigated whether preoperative 6MWD predicts the occurrence of 30-day PCs among patients undergoing VATS lobectomy for non-small-cell lung cancer. Our objective was to quantify the association between preoperative 6MWD and postoperative outcomes in a contemporary, minimally invasive surgical cohort and to consider its pragmatic utility for risk stratification and potential referral to perioperative prehabilitation pathways.

## 2. Materials and Methods

### 2.1. Participants

This study was a retrospective study of data collected from patient medical records between January and September 2022. The eligibility criteria were patients who were diagnosed with lung cancer and underwent VATS lobectomy at the University of Tsukuba Hospital. Patients were classified into PC and non-PC groups based on any complication within 30 days of surgery. Exclusion criteria included patients who underwent an intraoperative change in surgical approach from VATS to open thoracotomy and patients who did not have a record of their preoperative 6MWT.

### 2.2. Measurements

The investigated data, obtained from participants’ medical records, were age, sex, body mass index (BMI), comorbidities, medical history, surgical procedure, smoking history (Brinkman index [BI]), ECOG-performance status (ECOG-PS), preoperative 6MWT, preoperative respiratory function (spirometry), left ventricular ejection fraction (LVEF), laboratory tests: White blood cell (WBC) count, C-reactive protein (CRP) test, blood urea nitrogen (BUN), creatinine, aspartate aminotransferase (AST), alanine aminotransferase (ALT), lactate dehydrogenase (LDH), serum albumin (Alb) test, pathological stage of the cancer, and histological evaluation, whole tumor size, resected lobes, operative time, estimated blood loss. Handgrip strength was measured seated with the elbow at 90°, two maximal trials per hand after a practice trial and the best value from either hand was analyzed.

The 6MWT is a field walking test used to assess exercise tolerance. In this test, participants walk continuously back and forth along a 30 m flat and straight course, with the total distance walked over 6 min being measured [11,12]. The 6MWT followed a standardized corridor protocol (straight 30 m indoor course, timed verbal prompts) consistent with ERS/ATS technical standards. With reference to previous reports, 450 m was used as the cut-off point [13] by which participants were grouped into two groups: those ≤450 m as the low group and those ≥451 m as the high group. For respiratory function, preoperative forced vital capacity (FVC), forced expiratory volume in 1.0 s (FEV1.0), FEV1.0/FVC (FEV1.0%), and DLCO were investigated. The predicted values (%) were calculated from the standard regression equation of the Japanese Respiratory Society [14]. ECOG-PS is an index of general health and is used to determine the treatment strategy and efficacy of treatment for cancer [15]. It is defined on the standard 0–4 scale (0 = fully active; 1 = restricted in strenuous activity but ambulatory; 2 = ambulatory and capable of all self-care but unable to carry out any work activities; 3 = capable of only limited self-care, confined to bed/chair > 50% of waking hours; 4 = completely disabled). The pathological stages of cancer were classified into I to IV according to the international TNM Classification of Malignant Tumors (8th edition) [16,17]. Postoperative outcomes included pulmonary and non-pulmonary complications (overall), length of hospital stay (days), and days to ambulation (days). PCs were categorized as respiratory (pneumonia, atelectasis, ARDS, interstitial pneumonia exacerbation, prolonged air leak ≥ 5 days, bronchopleural fistula, pleuritis, ischemic bronchitis) or non-respiratory (atrial fibrillation, urinary tract infection, intestinal bleeding, and others).

### 2.3. Statistical Analysis

Regarding statistical analysis, the Mann–Whitney U test was used for continuous variables in the two-group comparison, and the χ^2^ test was used for categorical variables. For multivariate analysis, logistic regression analysis was performed with the items that showed significant differences in the two-group comparison as explanatory variables. The dependent variable was set at 1 for the group with complications and 0 for the group without complications, and the forced entry method was used. Logistic regression was applied to identify predictors of PCs. Variables showing significant differences between groups were included as covariates in the multivariable analysis. The multivariable analysis was limited to four covariates that showed significant differences between the two groups, in order to reduce overfitting, given the small number of events.

Receiver-operating characteristic (ROC) analysis was performed to evaluate the discriminative ability of preoperative 6MWD for 30-day PCs. The area under the curve (AUC) was estimated using a non-parametric approach. An optimal cut-off value was derived from the ROC curve without pre-specifying a threshold by maximizing the combined sensitivity and specificity. Missing data (<5%) were imputed by mean substitution. Multicollinearity was evaluated using variance inflation factors (VIF < 2 for all variables). ROC curve analysis was used to determine optimal 6MWD cut-off values based on the Youden index, derived from SPSS output (‘Coordinates of the Curve’ table). SPSS Statistics Ver. 27 (IBM Corporation, Tokyo, Japan) was used for the analysis. Significance level was set at less than 5% two-sided.

### 2.4. Ethical Considerations

This study was conducted under the approval of the Clinical Research Ethics Committee of the University of Tsukuba Hospital (Approval number: R02-107). An opt-out consent approach was applied in accordance with institutional policy.

## 3. Results

Eighty-one participants who underwent VATS lobectomy at our hospital within the study period were included. Of those 83 participants, 17 patients were excluded from the study: 3 participants for intraoperative change in surgical approach from VATS to open thoracotomy and 12 for having no record of preoperative 6MWT. Excluded patients did not differ significantly in baseline characteristics from those patients. Redo surgery cases were excluded. The remaining 66 patients were categorized into two groups: 11 participants with pulmonary complications (PC Group) and 55 without pulmonary complications (Non-PC Group). Postoperative outcomes included pulmonary and non-pulmonary complications (overall).

Comparison of basic attributes between the two groups showed no significant differences in age, sex, smoking index (BI), respiratory function, ECOG-PS, LVEF, whole tumor size, resected lobes, operative time, estimated blood loss and cancer stage (pathological stage) (Table 1A,B). The breakdown of PCs was: pneumonia (*n* = 3), atelectasis (*n* = 1), ARDS (*n* = 1), lung fistula (*n* = 2), exacerbation of interstitial pneumonia (*n* = 2), bronchopleural fistula (*n* = 1), pleuritis (*n* = 1), ischemic bronchitis (*n* = 1), AF (*n* = 7), urinary tract infection (*n* = 1), intestinal bleeding (*n* = 1), and others (*n* = 6). Multiple responses were allowed, and a total of 11 patients experienced postoperative respiratory complications (Table 2).

**Figure 1:**
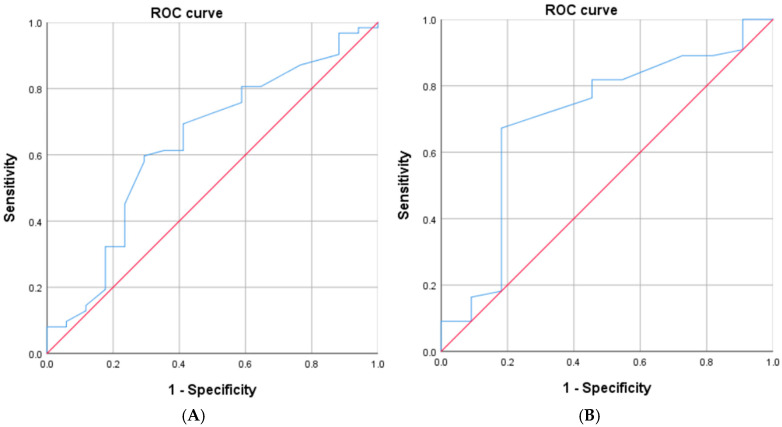
(**A**) Receiver operating characteristic (ROC) curve of the 6-minute walk test (6MWT) for postoperative overall complications. Data-driven optimal cut-off (Youden index) at 450 m with sensitivity 0.694 and specificity 0.588; area under the curve (AUC) 0.636. (**B**) ROC curve of the 6-minute walk test (6MWT) for postoperative pulmonary-related complications. Data-driven optimal cut-off (Youden index) at 450 m with sensitivity 0.673 and specificity 0.818; AUC 0.703.

In the comparison of the two groups, significant differences were observed in BMI, preoperative 6MWT (≤450 m), and preoperative BUN. In the multivariate analysis, there was a significant difference in the preoperative 6MWT (≤450 m) (OR: 5.674, 95% CI: 1.206–26.684, *p* = 0.028) and the length of hospital stay (OR = 1.856, 95% CI = 1.004–3.434, *p* = 0.049). There were no significant differences in BMI, BUN, and CRP (Table 3A,B). The 6MWD cut-off point was determined by ROC curve analysis. Without pre-specifying a threshold, the data-driven optimal cut-off value was set at 450 m, with a sensitivity of 0.694 and specificity of 0.588 for overall complications, and a sensitivity of 0.673 and specificity of 0.818 for pulmonary complications (Figure 1A,B). For overall complications (Figure 1A), the AUC was 0.636 (SE = 0.078, *p* = 0.088, 95% CI: 0.482–0.789), whereas for pulmonary complications (Figure 1B), it was 0.703 (SE = 0.093, *p* = 0.034, 95% CI: 0.521–0.885), with the same optimal cut-off value (450 m; determined using the Youden index). No patients received neoadjuvant therapy.

## 4. Discussion

The present study demonstrated that a preoperative 6MWD of ≤450 m was an independent predictor of postoperative pulmonary complications in patients undergoing VATS lobectomy for non-small-cell lung cancer. The predictive ability of the 6MWD was moderate for overall complications (AUC = 0.636) but improved when focusing on pulmonary complications (AUC = 0.703). In addition, the logistic regression analysis revealed that the length of hospital stay was also significantly associated with pulmonary complications. These findings suggest that preoperative functional capacity, as measured by the 6MWT, and postoperative recovery indicators such as hospital stay are both closely linked to the risk of respiratory morbidity following VATS lobectomy. Preoperative 6MWT ≤ 450 m was extracted as a significant predictor of PC in the multivariate analysis (odds ratio: 5.674, 95% CI: 1.206–26.684, *p* = 0.028; Table 3B). This result aligns with previous studies that suggest preoperative exercise capacity is associated with postoperative prognosis in thoracic surgery. Notably, Santos et al. reported that preoperative 6MWD predicted postoperative pneumonia following open thoracotomy for lung cancer [18]. Their findings emphasized the utility of 6MWD as a marker of pulmonary functional reserve and respiratory muscle strength.

The present study shares common ground with Santos et al. in that both identify the preoperative 6MWT as a simple, non-invasive, and valuable tool in predicting postoperative complications, particularly pulmonary complications such as pneumonia and atelectasis [18,19]. However, there are several important differences between the two studies. First, Santos et al. focused on patients undergoing open thoracotomy, whereas the current study examined patients undergoing VATS lobectomy, a far less invasive approach. The physiological stress and respiratory impact differ significantly between open thoracotomy and VATS, yet the predictive value of the 6MWT appears to be retained in both. This strongly suggests the robustness of the 6MWT across surgical modalities [5,6,17]. Second, while Santos et al. emphasized pneumonia as the primary complication, our study broadened the scope to include a comprehensive list of complications, such as acute respiratory distress syndrome (ARDS), bronchopleural fistula, atrial fibrillation (AF), and urinary tract infections (UTI), providing a more comprehensive view of postoperative risk (Table 2). Our multivariate analysis demonstrated that low 6MWT is not only associated with pulmonary complications but also with a wider spectrum of adverse events following surgery (Table 3). Third, the cut-off value of 450 m, adopted in the present study, contributes to clinical practicality. Although previous literature has reported various thresholds for 6MWT (e.g., 400 m, 500 m), our results confirm that ≤450 m can serve as a useful reference point for risk stratification in patients scheduled for VATS lobectomy [9,10,18]. This cutoff value was selected based on prior studies demonstrating its clinical significance in predicting postoperative outcomes [13], in addition to utilizing cutoff values obtained from ROC analysis (Figure 1). In previous studies of patients undergoing lung resection, a preoperative 6MWD of ≤450 m has been reported as a threshold associated with an increased risk of postoperative pneumonia [20]. This finding serves as one of the rationales for using 450 m as the cut-off value in the present study.

Collectively, these distinctions highlight the novelty of the present study. Furthermore, to our knowledge, few reports have examined the association between preoperative 6MWT and complications specifically in VATS lobectomy cases. Given the growing prevalence of VATS in clinical practice, identifying a simple preoperative indicator applicable to this surgical population fills an important gap in the literature.

From a physiological perspective, patients with reduced 6MWT distances may exhibit impaired cardiopulmonary reserve, reduced diaphragmatic function, or decreased muscular endurance. These factors can limit effective postoperative coughing, impede sputum clearance, and delay early mobilization, which are mechanisms known to contribute to PC such as atelectasis and pneumonia [7,21,22]. Therefore, implementing preoperative rehabilitation programs aimed at improving walking endurance may contribute to reducing postoperative morbidity.

Our findings demonstrate that a pre-specified 6MWD threshold of 450 m independently identifies patients at higher risk of 30-day PCs after VATS lobectomy. This is consistent with prior reports identifying ~500 m as discriminative with internal validation [22,23,24] and with respiratory-specific outcomes linked to lower 6MWD [25]. Recent evidence from uniportal VATS suggests optimal cut-offs around ~458 m within a pre-habilitation program [26], aligning with our choice of a clinically actionable 450 m threshold.

Physiologically, shorter 6MWD likely reflects reduced cardiopulmonary reserve and respiratory muscle performance, limiting early mobilization and sputum clearance and predisposing to pulmonary morbidity. Using a comprehensive morbidity endpoint may dilute pulmonary-specific effects; nevertheless, the direction and magnitude of the association were consistent. Pulmonary-specific analyses and exploration of perioperative pre-habilitation, particularly for patients walking ≤450 m, are warranted, given randomized evidence that a 2-week multimodal program improves perioperative 6MWD after VATS lobectomy [27] and contemporary thoracic rehabilitation overviews [28]. The predictive ability of 6MWD was moderate for overall complications (AUC = 0.636) but improved for pulmonary complications (AUC = 0.703), which supports its clinical relevance for respiratory outcomes. These results align with prior reports emphasizing the role of exercise tolerance in predicting postoperative morbidity. Although the AUC was below 0.70 for all events, the improvement to 0.703 for pulmonary complications suggests that 6MWD has greater discriminative value when focusing on respiratory-related events. The optimal 6MWD cut-off identified by ROC analysis was 450 m in both analyses, corresponding to the maximum Youden index. This consistent threshold reinforces its robustness as a clinical indicator of perioperative fitness.

The spirometry results (FVC and FEV1.0) did not differ significantly between the two groups and were not identified as independent predictors in the multivariate analysis. This may be explained by the fact that spirometric parameters mainly reflect static ventilatory function, whereas the 6MWT captures integrated cardiopulmonary and musculoskeletal performance, including oxygen transport, cardiovascular response, and peripheral muscle endurance. Therefore, the 6MWT may better represent the overall physiological reserve and perioperative functional capacity of patients than spirometry alone. Moreover, most patients in this cohort had relatively preserved preoperative lung function (mean FEV1.0% > 70%), which could have caused a ceiling effect that limited the discriminative power of spirometric variables. These factors likely explain why 6MWT, but not spirometry, showed a significant association with postoperative complications.

In the logistic regression analysis focusing on pulmonary complications, the length of hospital stay was also identified as a significant factor. This finding suggests that prolonged hospitalization may reflect postoperative complications that delay recovery, or conversely, that patients with greater frailty or impaired physiological reserve are more likely to experience both longer hospital stays and pulmonary complications. Therefore, the relationship between 6MWT performance and hospital stay duration may indicate that reduced preoperative functional capacity contributes to delayed postoperative recovery. These results are consistent with the latest ERS/ESTS guidelines on preoperative fitness assessment (2023) [29], which recommend incorporating functional exercise tests such as the 6MWT to evaluate perioperative risk in patients undergoing lung resection.

The results of the two-group comparison (Table 1A) showed that BUN and CRP levels were significantly higher in the group with complications (PC Group) than in the group without complications (Non-PC Group). According to previously published literature, BUN can be used as a predictor of fluid volume and fluid status in older patients, and elevated BUN levels have been reported to be associated with in-hospital mortality in urinary tract infections in this population [30]. Fluid status is closely related to organ function, immune response, and inflammatory processes, all of which can influence postoperative outcomes [31]. Similarly, CRP is regarded as an indicator of systemic inflammation, with elevated levels reflecting stress or an invasive procedure that may lead to a decline in general condition [32]. Elevated preoperative CRP levels may also reflect advanced cancer stages. However, in the current study, BUN and CRP values were within the normal range in both groups and were not identified as significant predictors in the logistic regression analysis (Table 3A), suggesting that these indicators alone may not sufficiently explain the risk of PC in this population. This may be partly attributable to the small number of patients with advanced-stage cancer in our cohort. Regarding the model specification, the multivariable analysis was limited to four covariates that showed significant differences between the two groups, in order to reduce overfitting given the small number of events.

This study has several limitations. It was conducted at a single institution with a relatively small sample size, which limits the generalizability of the findings. Additionally, its retrospective design may introduce selection bias. Nevertheless, the identification of preoperative 6MWT as a predictor of postoperative complications in VATS lobectomy patients adds clinically relevant insight, especially in the context of minimally invasive surgery where risk factors may differ from traditional thoracotomy.

## 5. Conclusions

In summary, the results of our study suggest that a preoperative 6MWD ≤ 450 m can be used to identify patients at increased risk of postoperative complications after VATS lobectomy for NSCLC. While these findings are preliminary, and future research employing more robust statistical methods is warranted for validation, we can nevertheless assert that for patients undergoing VATS lobectomy for lung cancer, the preoperative 6MWT is certainly a useful predictive indicator of PCs. In various clinical settings, this simple measure can complement standard spirometry to support perioperative risk stratification and pre-habilitation referral.

## Figures and Tables

**Table 1:** (**A**) Demographic information of patients (postoperative overall complications) and (**B**) demographic information of patients (postoperative pulmonary-related complications).

(**A**)			
**Characteristics**	**PC Group (*n* = 20)**	**Non-PC Group (*n* = 46)**	***p***
	***n* (%)**	***n* (%)**	
Age	73 ± 4	68 ± 10	0.142
Sex, Female (%)	8 (40%)	20 (43%)	0.364
Male (%)	12 (60%)	26 (57%)	
BMI (kg/m^2^)	25 ± 4	23 ± 4	0.018 *
Brinkman index (smoking)	898 ± 770	490 ± 271	0.143
Comorbidity			0.269
COPD	2 (10%)	2 (4%)	
Interstitial pneumonia	2 (10%)	1 (2%)	
Hypertension	12 (60%)	19 (41%)	
Diabetes mellitus	5 (25%)	7 (15%)	
CRP (mg/dL)	0.4 ± 0.7	0.1 ± 0.3	0.048 *
BUN (mg/dL)	17 ± 4	14 ± 4	0.002 **
WBC (μL)	6615 ± 1867	6043 ± 1580	0.258
Alb (g/dL)	3 ± 0.4	4 ± 0.4	0.136
FVC (% predicted)	116 ± 17	108 ± 23	0.128
FEV1.0 (%predicted)	101 ± 22	95 ± 26	0.549
FEV1.0 %	71 ± 9	73 ± 10	0.402
DLCO (% predicted)	102 ± 34	105 ± 34	0.686
LVEF	51 ± 12	53 ± 15	0.257
Histology Adenocarcinoma	12 (60%)	33 (71%)	0.608
Squamous cell Carcinoma	6 (30%)	10 (22%)	
Others	2 (10%)	3 (7%)	
Grip strength (kg)	30 ± 11	26 ± 9	0.126
6MWT (m)	454 ± 65	503 ± 77	0.011 *
Days to ambulation (days)	6 ± 11	3 ± 3	0.341
Length of hospital stay (days)	11 ± 13	9 ± 1	<0.01 **
ECOG-PS: 0	17 (85%)	44 (95%)	0.397
1	3 (15%)	2 (5%)	
2, 3, 4	0 (0%)	0 (0%)	
Whole tumor size (mm)	32 ± 14	29 ± 13	0.118
Resected lobes Upper Middle Lower	9 (45%) 0 (0%) 11 (55%)	23 (50%) 2 (4%) 21 (46%)	0.551
Operative time (min)	223 ± 62	293 ± 447	0.346
Estimated blood loss (mL)	56 ± 78	70 ± 165	0.835
Pathological stage			0.463
IA·IB	13 (65%)	32 (70%)	
IIA~IIIB	7 (35%)	14 (30%)	
(**B**)			
**Characteristics**	**PC Group (*n* = 11)**	**Non-PC Group (*n* = 55)**	***p***
	***n* (%)**	***n* (%)**	
Age	72 ± 5	70 ± 10	0.959
Sex, Female (%)	4 (36%)	24 (44%)	0.482
Male (%)	7 (64%)	31 (56%)	
BMI (kg/m^2^)	26 ± 5	23 ± 3	0.027 *
Brinkman index (smoking)	950 ± 1137	547 ± 675	0.216
Comorbidity			0.269
COPD	1 (9%)	3 (5%)	
Interstitial pneumonia	2 (17%)	1 (1%)	
Hypertension	8 (70%)	23 (42%)	
Diabetes mellitus	3 (22%)	9 (16%)	
CRP (mg/dL)	0.7 ± 0.2	0.6 ± 0.2	0.294
BUN (mg/dL)	17 ± 4	15 ± 4	0.046
WBC (μL)	6691 ± 1839	6022 ± 3085	0.095
Alb (g/dL)	4 ± 0.3	4 ± 0.5	0.511
FVC (% predicted)	114 ± 12	109 ± 23	0.530
FEV1.0 (%predicted)	103 ± 19	96 ± 26	0.294
FEV1.0 %	74 ± 8	72 ± 10	0.282
DLCO (% predicted)	90 ± 36	107 ± 33	0.111
LVEF	50 ± 11	53 ± 15	0.468
Histology Adenocarcinoma	7 (64%)	38 (69%)	0.237
Squamous cell Carcinoma	3 (27%)	13 (23%)	
Others	1 (9%)	4 (8%)	
Grip strength (kg)	30 ± 10	27 ± 9	0.234
6MWT (m)	449 ± 77	496 ± 75	0.034
Days to ambulation (days)	7 ± 14	3 ± 2	0.732
Length of hospital stay (days)	14 ± 23	9 ± 2	<0.01 **
ECOG-PS: 0	8 (73%)	53 (96%)	0.199
1	3 (27%)	2 (4%)	
2, 3, 4	0 (0%)	0 (0%)	
Whole tumor size (mm)	35 ± 15	30 ± 13	0.245
Resected lobes Upper Middle Lower	3 (27%) 0 (0%) 8 (73%)	29 (53%) 2 (4%) 24 (43%)	0.199
Operative time (min)	243 ± 74	278 ± 410	0.624
Estimated blood loss (mL)	69 ± 101	65 ± 152	0.812
Pathological stage			0.287
IA·IB	6 (55%)	39 (71%)	
IIA~IIIB	5 (45%)	16 (29%)	

* *p* < 0.05; ** *p* < 0.01; Mean ± SD; *n* (%); Mann–Whitney U test; χ^2^ test. PC, postoperative complications; BMI, body mass index; COPD, chronic obstructive pulmonary disease; CRP, C reactive protein; BUN, blood urea nitrogen; Alb, Serum Albumin; FVC, forced vital capacity; FEV1.0, forced expiratory volume at 1.0 s; LVEF, left ventricular ejection fraction; 6MWT, 6-minute walk test; ECOG-PS, Eastern Cooperative Oncology Group-performance status.

**Table 2:** Types of postoperative complications.

	Patients (N = 66) *n* (%)
Pneumonia	3 (4.5%)
Atelectasis	1 (1.5%)
ARDS	2 (3.0%)
Lung fistula	2 (3.0%)
IP exacerbation	2 (3.0%)
Bronchopleural fistula	1 (1.5%)
Pleuritis	1 (1.5%)
Ischemic bronchitis	1 (1.5%)
AF	7 (10.6%)
Urinary tract infection	1 (1.5%)
Intestinal bleeding	1 (1.5%)
Others	6 (9.0%)

ARDS, acute respiratory distress syndrome; IP, interstitial pneumonia; AF, atrial fibrillation. Multiple responses were allowed.

**Table 3:** (**A**) Factors related to the postoperative overall complications for patients. (**B**) Factors related to the postoperative pulmonary-related complications for patients.

(**A**)			
**Independent Variables**	**Odds Ratio**	**95% CI**	***p***
BUN	1.187	1.000–1.409	0.050
6MWT (≤450 m) ^†^	4.765	1.307–17.366	0.018 *
6MWT (m) ^†^	0.992	0.984–1.001	0.096
BMI	1.164	0.964–1.404	0.114
CRP	1.968	0.638–6.069	0.239
(**B**)			
**Independent Variables**	**Odds Ratio**	**95% CI**	***p***
BUN	1.387	0.514–1.155	0.066
6MWT (≤450 m) ^†^	5.674	1.206–26.684	0.028 *
6MWT (m) ^†^	0.996	0.981–1.011	0.601
BMI	1.146	0.913–1.438	0.241
Length of hospital stay (days)	1.856	1.004–3.434	0.049 *

* *p* < 0.05; Mean ± SD; *n* (%); CI, confidence interval; BUN, blood urea nitrogen; BMI, body mass index; CRP, C reactive protein; 6MWT, 6-minute walk test. ^†^: The variables, 6MWT (≤450 m) and 6MWT (m), were entered separately into the logistic regression model (analyzed as independent models) to avoid multicollinearity.

## Data Availability

The original contributions presented in the study are included in the article; further inquiries can be directed to the corresponding author/s.
